# Clinical Outcome Following Surgical Repair of Small Versus Large Orbital Floor Fractures Using Polyglactin 910/Polydioxanone (Ethisorb^®^)

**DOI:** 10.3390/ma13010206

**Published:** 2020-01-03

**Authors:** Otto Steinmassl, Johannes Laimer, Vincent Offermanns, Matthias Wildauer, Patricia-Anca Steinmassl, Astrid E. Grams, Ferdinand Kofler, Michael Rasse, Emanuel Bruckmoser

**Affiliations:** 1 University Hospital for Cranio-Maxillofacial and Oral Surgery, A-6020 Innsbruck, Austria; 2 University Hospital for Radiology, Medical University of Innsbruck, A-6020 Innsbruck, Austria; 3 University Hospital for Dental Prosthetics and Restorative Dentistry, A-6020 Innsbruck, Austria; 4 University Hospital for Neuroradiology, A-6020 Innsbruck, Austria; 5 Private Practice for Oral and Maxillofacial Surgery, A-5020 Salzburg, Austria

**Keywords:** orbital floor fracture, reconstruction, polyglactin 910/polydioxanone, Ethisorb^®^

## Abstract

The aim of this retrospective study was to evaluate the clinical outcome of surgical management of small versus large, isolated orbital floor fractures (OFFs) using polyglactin 910/polydioxanone (Ethisorb^®^). Covering a four-year period (2010–2013), all records concerning midfacial fractures with involvement of the orbit were screened. Isolated fractures of the orbital floor as well as combined injuries of the orbital floor and medial wall that had been treated surgically using polyglactin 910/polydioxanone (Ethisorb^®^) were included. Patients underwent a preoperative, a postoperative, and a late ophthalmologic assessment. The clinical outcomes of surgically managed small OFFs up to 2 cm^2^ were statistically analyzed and compared to clinical results in larger defects. The final sample included 61 patients (25 women, 36 men). Fractures up to 2 cm^2^ were found in 33 patients (54.1%), whereas 28 patients (45.9%) suffered from OFFs larger than 2 cm^2^. The clinical outcomes did not significantly differ between both sample categories, and statistical analysis showed a power of 0.91 to detect a potentially existing difference. On final examination, 52 patients were free of any clinical symptoms, whereas minor issues were found in seven subjects, and two patients suffered from severe impairment. In conclusion, polyglactin 910/polydioxanone (Ethisorb^®^) seems to be a suitable material for surgical repair of both small and large OFFs.

## 1. Introduction

Fractures of the orbital floor can be isolated as so-called ‘blow-out fractures’ or occur in combination with other midfacial injuries. Different theories have addressed the pathogenetic mechanisms leading to isolated orbital floor fractures (OFFs). In one theory, this type of injury was described as being the result of force transmission from the rigid infraorbital rim to the weak orbital floor [1]. In another theory, it was proposed that hydraulic pressure from the globe would be transmitted to the thin orbital floor causing it to break [2]. The current opinion regarding this controversy is that both mechanisms can produce blow-out fractures, but with different characteristics [3]. Finally, the two mechanisms can also act in combination resulting in OFFs of significantly larger size [4].

Clinically, OFFs present with typical signs and symptoms including periocular swelling, subconjunctival hemorrhage, ecchymosis, chemosis, proptosis, enophthalmos, diplopia, and hypoesthesia of the infraorbital nerve [5]. Since associations were found between horizontal diplopia and medial wall fractures as well as between diplopia on upgaze and orbital floor fractures, these clinical findings could be helpful indicators in the assessment of medial wall and orbital floor fractures, respectively [6]. As the rate of acute visual loss in fractures involving the orbit lies around an average of 1.7 percent [7], a prompt ophthalmologic examination is imperative. Apart from assessing visual acuity, the ophthalmologic and orthoptic status will also include evaluation of globe integrity, intraocular pressure, cornea, retina, eyeball motility, and perimetric readings.

Radiologically, OFF diagnosis is mainly based on coronal and sagittal computed tomography (CT) views allowing for assessment of both bony and soft tissue structures including the external eye muscles. Special importance has to be attached to the inferior rectus muscle since entrapment of this structure in so called trap-door fractures requires early surgical intervention in symptomatic cases [8]. Although conventional computed tomography can still be regarded as the gold standard for orbital trauma imaging, cone beam CT has also been shown to depict OFFs with remarkably high sensitivity and specificity [9].

Clinical management of OFFs is initially based on conservative measures including analgesia and anticongestive nose drops or sprays as needed. The use of antibiotics is controversial, and routine administration of such drugs as standard treatment does not seem to be justified [10]. However, a postoperative one-day course of antibiotics is advocated in displaced orbital fractures [11]. The most important instruction for the patient to be followed is not to blow the nose as this can cause significant emphysema in the facial region. For none or barely dislocated OFFs without enophtalmus, diplopia, or impaired infraorbital nerve sensibility, there is generally no need for further treatment other than conservative measures. However, clinical follow-up examinations are recommended in the first weeks, if not months.

Apart from indications for immediate surgical repair including non-resolving oculocardiac reflex, trap-door fractures, and early enophthalmos or hypoglobus [12], operative interventions are usually delayed for several days until the swelling subsides. Furthermore, the patient can then be reevaluated to determine definitively the necessity for an operative OFF repair. With regard to the timing of surgery, in most cases a two-week window is common practice. The incidence of diplopia, enophthalmos, and infraorbital nerve dysfunction was shown to be significantly lower in patients undergoing surgery within two weeks of the trauma [13]. However, other evidence can be found, too, reporting no outcome difference between early (within two weeks) and late surgical repair [14].

When it comes to reconstructing the orbital floor, there is a great variety of different materials which have been described and summarized comprehensively in a systematic review [15] some years ago. The following enumeration refers to this review. Autogenous materials for this indication comprise autologous bone (calvarial bone, iliac bone, nasal septal bone, maxillary antral bone, and mandibular bone), nasoseptal or conchal auricular cartilage, and temporalis fascia. Allogeneic materials include lyophilized human dura mater, irradiated homologous fascia lata, and irradiated cartilage. The use of collagen membranes of porcine origin as xenograft material was reported in one study [16]. Alloplastic materials used for OFF repair can be classified as resorbable or nonresorbable. The latter category comprises titanium meshes or plates, porous polyethylene, bioactive glass, and hydroxyapatite. Resorbable alloplastic materials include pure polymerized poly-L-lactide (PLLA) and its derivatives, poly-L/DL-lactide (P[L/DL]LA 70/30), PLLA with polyglycolic acid (PGA), self-reinforced polyglycolic acid membranes (SRPM), polydioxanone (PDS), and polyglactin 910/polydioxanone (Ethisorb^®^) which will be described in more detail in the following two paragraphs.

Ethisorb^®^ (Ethicon, Norderstedt, Germany) is a synthetic, resorbable material consisting of undyed Vicryl (Polyglactin 910) and undyed polydioxanone (PDS). The different melting points of these components allow for a thermoplastic cross-interlocking resulting in a composite material with a 3-dimensional filamentary structure forming the framework for endogenous connective tissue [17]. Differing resorption times of both components result in a graded hydrolysis of the whole complex, whereby Polyglactin 910 will dissolve within 45–60 days and polydioxanone within approximately 90–180 days [18].

Ethisorb^®^ was originally described as an alloplastic material suitable for closure of dura mater defects in neurosurgery [19,20] and has proven to be of great value in this indication [21]. However, off-label use of this material for the repair of OFFs has been described, too [22,23,24,25]. With regard to the fact that this material is not licensed for the use in orbital surgery it was the aim of our retrospective clinical study to further assess its value in OFF repair with a focus on ophthalmologic outcome. In particular, we wanted to determine if Ethisorb^®^ can be a valuable treatment option for the surgical repair of both small and large OFFs.

## 2. Materials and Methods

For this retrospective analysis, clinical records of all patients with midfacial fractures involving the orbit who were surgically treated at the University Hospital for Cranio-Maxillofacial and Oral Surgery, Innsbruck, Austria, between January 2010 and December 2013 were screened. The study has been approved by the Ethics Committee of the Medical University of Innsbruck (reference number: AN2017-0059 371/4.14; date of approval: 24.11.2017) and was carried out following the rules of the Declaration of Helsinki of 1975, revised in 2013.

The detailed inclusion criteria were as follows: isolated fracture of the orbital floor with or without involvement of the medial wall, surgical management of the injury using Ethisorb^®^, OP notes with detailed information regarding surgical procedure and reconstruction method, preoperative CT scan including coronal views, pre- and postoperative ophthalmologic and orthoptic exams. Exclusion criteria comprised zygomaticoorbital and complex midfacial fractures, isolated fractures of the medial orbital wall or the orbital roof, limited ophthalmologic examination due to intubation, and fracture repairs without use of Ethisorb^®^ or with additional reconstructive materials or techniques, such as packing of the maxillary sinus. Cases lacking information about the clinical long-term follow-up examination were also excluded from the analysis.

All preoperative CT scans were evaluated by an experienced radiologist. The area of the orbital defect was measured, and the orbital fracture was classified according to Jaquiéry [26]. In this classification, category I refers to isolated defects of the orbital floor or the medial wall measuring between one and two square centimeters. In category II defects, fractures of the orbital floor and/or medial wall exceed two square centimeters whereby the bony ledge at the medial margin of the infraorbital fissure is preserved. In category III defects (fractures exceeding two square centimeters) this bony ledge is missing. Defects of the entire orbital floor and the medial wall extending into the posterior third with missing bony ledge are classified as category IV. The last group of injuries (category V) refers to fractures extending into the orbital roof.

Regarding clinical assessment, all patients underwent a thorough ophthalmologic examination on the day of admission to rule out severe damage to the eyeball that would require immediate surgery. Following decline of the usually present concomitant periorbital swelling, subjects were re-assessed by an ophthalmologist preoperatively including a detailed orthoptic examination. Postoperatively, all patients underwent ophthalmologic and orthoptic assessment again. Further examinations of that kind were scheduled as needed in case of pathologic features present at the postoperative reevaluation assessment. Patients were followed clinically in regular intervals up to one year after the trauma. According to the outcome of the respective orthoptic examination, patients were grouped into 3 categories. Patients in category “no impairment” would not show any abnormalities at all. Subjects classified in category “mild impairment” had minor pathological features comprising diplopia outside the usual field of view or malposition of the eyeball without functional impairment in daily life activities. Finally, in the category “severe impairment”, severe abnormalities were noted including persistent diplopia or malposition of the eyeball affecting daily life activities.

The data was analyzed using SPSS Statistics 24 (IBM, Armonk, NY, USA) and R 3.3.1 (R Foundation for statistical computing, Vienna, Austria). Continuous measures were described as mean values and standard deviations (SD), categorical data as absolute and relative frequencies (absolute numbers and percentages).

The non-parametric Friedman test was used to determine potential differences in functional ophthalmologic findings between the preoperative, early postoperative and late postoperative examinations. Pairwise comparisons were performed applying a Bonferroni correction for multiple comparisons.

To determine whether there were statistically significant differences concerning functional ophthalmologic findings between patients with small and large orbital floor fractures according to Jaquiéry’s classification, the Mann–Whitney U test was employed.

The significance level for all statistical tests was set at α = 0.05. In order to assess the statistical power for detecting differences between the two groups, a post hoc power analysis was performed using the software G*Power 3.1 (Heinrich Heine University, Düsseldorf, Germany).

## 3. Results

Between January 2010 and December 2013, a total number of 952 patients with midfacial fractures involving the orbit were treated at the University Hospital for Cranio-Maxillofacial and Oral Surgery, Innsbruck, Austria. Of these, 567 suffered from zygomaticoorbital, 81 from complex midfacial, and 304 from isolated orbital wall fractures. In the latter group, 225 patients were treated conservatively, whereas 79 subjects underwent surgical repair. Fractures concerning the orbital roof alone (3 patients) or in combination with the orbital floor (2 patients) were not included in this study. Surgically treated isolated medial wall fractures (6 patients) were also excluded from the study.

An isolated fracture of the orbital floor with or without involvement of the medial wall was diagnosed in 68 patients. In the majority (92.6%) of these cases, Ethisorb^®^ was used for surgical reconstruction, PDS foils were used in 4 cases, and in 1 patient the bony fragments of the orbital floor were repositioned without application of any foreign material. No patient in this group was treated using a titanium mesh. Patients not treated with Ethisorb^®^ were excluded from the study as well as 2 cases with missing information about the clinical long-term follow-up examination.

With regard to the protocol of our retrospective analysis, 61 patients met the predefined criteria and were therefore included in the present study.

There were 25 (41.0%) women and 36 (59.0%) men. The mean age in women was 53.9 years (range: 13.4 to 87.1 years), whereas men were aged 38.1 years on average (range: 8.1 to 74.4 years). The most common causes of injury included sports accidents (37.7%), falls (23.0%), and interpersonal violence (21.3%), followed by traffic accidents (8.2%), accidents at work (3.3%), and miscellaneous other (6.6%).

49 (80.3%) of the 61 surgically managed orbital wall fractures were pure orbital floor fractures. In 12 patients (19.7%), both the orbital floor and the medial wall were affected.

According to the Jaquiéry classification, more than half of the injuries (33 patients, 54.1%) were classified as category I fractures. The remainder were ranked as categories II (20 patients, 32.8%), III (7 patients, 11.5%), and IV (1 patient, 1.6%) defects, respectively. The mean size of the defect with the respective standard deviation was 0.82 cm^2^ (SD = 0.68) in class I, 2.88 cm^2^ (SD = 0.60) in class II, and 2.68 cm^2^ (SD = 0.59) in class III defects. The defect size of the class IV defect in one patient was 4.6 cm^2^.

**Figure 1:**
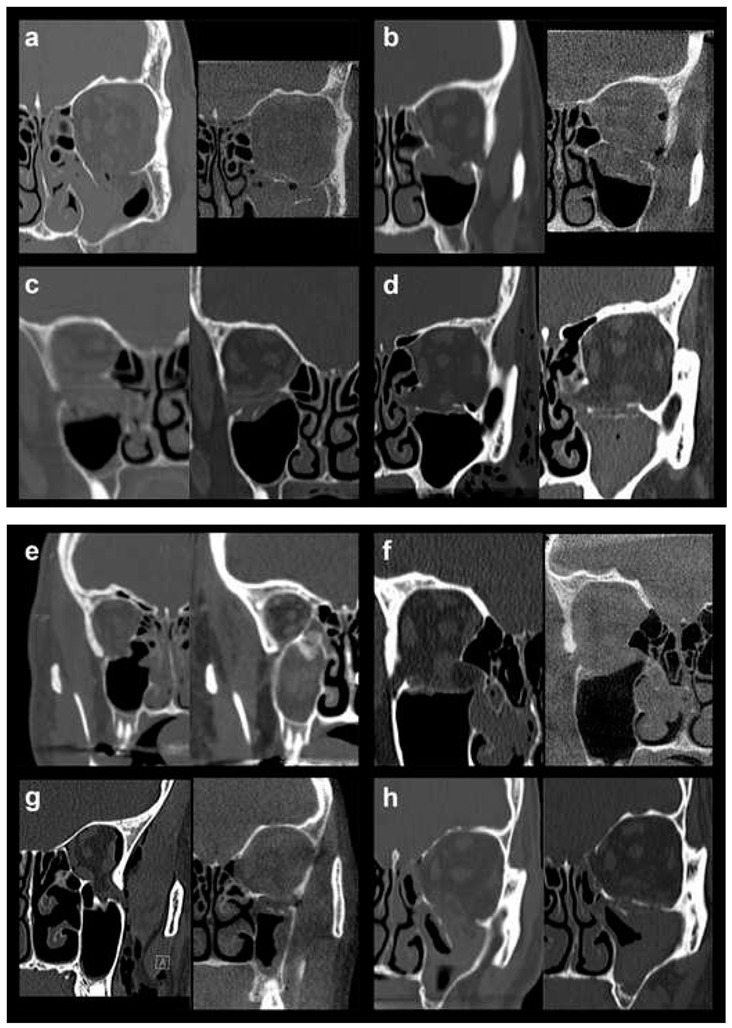
Pre- and postoperative conventional or cone-beam CT scans (coronal views) of all 7 class III fractures (**a**–**g**) and 1 class IV fracture (**h**).

In order to allow for a valid statistical comparison between small and large defects, OFFs comprising Jaquiéry classes II to IV were merged into the “large defect” OFF group (28 patients, 45.9%). This collective category was statistically compared to the “small defect” OFF group represented by injuries classified as Jaquiéry I (33 patients, 54.1%), thus, up to 2 cm^2^. In Figure 1, pre- and postoperative conventional or cone-beam CT scans (coronal views) of all 7 class III fractures (Figure 1a–g) and 1 class IV fracture (Figure 1h) are shown.

**Figure 2:**
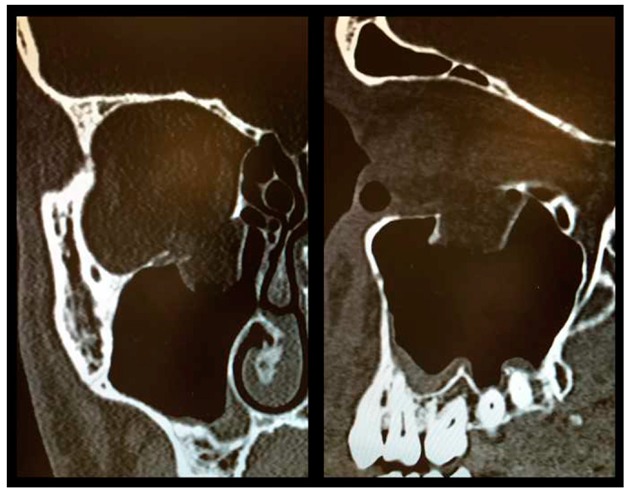
Preoperative CT scan showing a large OFF ((**left**) coronal view, (**right**) sagittal view).

**Figure 3:**
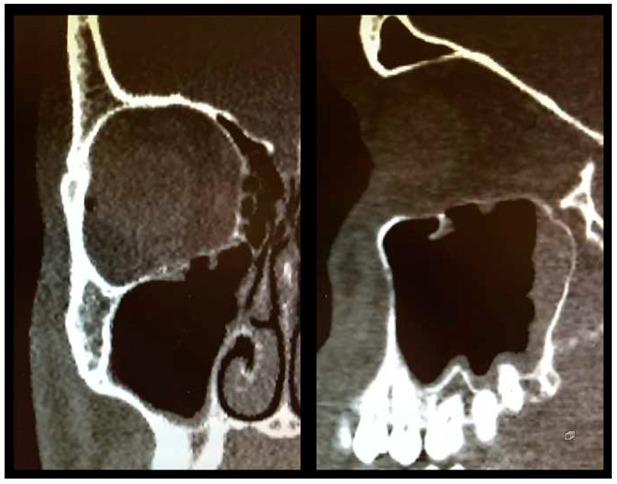
Postoperative CT scan following OFF reduction and placement of Ethisorb^®^ ((**left**) coronal view, (**right**) sagittal view).

As described above, all patients underwent a first ophthalmologic examination on the day of admission. Following the decline of the frequently present periorbital swelling, all patients had a detailed orthoptic assessment preoperatively. The mean time elapsed from injury to the detailed orthoptic assessment was 1.93 days (SD = 1.45; range: 0–7 days). The mean time between the day of injury and the surgical repair using Ethisorb^®^ patches was 4.33 days (SD = 2.71; range: 0–13 days). A representative example of a typical case is illustrated in Figure 2 and Figure 3 showing both the pre- and postoperative CT scans.

All patients underwent a second orthoptic assessment postoperatively which took place 3.7 days following surgery (SD = 4.5; range: 0–23 days postoperatively). All patients showing pathological features in this postoperative examination were invited to attend one final orthoptic assessment which they had 48.6 days post surgery (SD = 51.6; range: 6–163 days).

**Figure 4:**
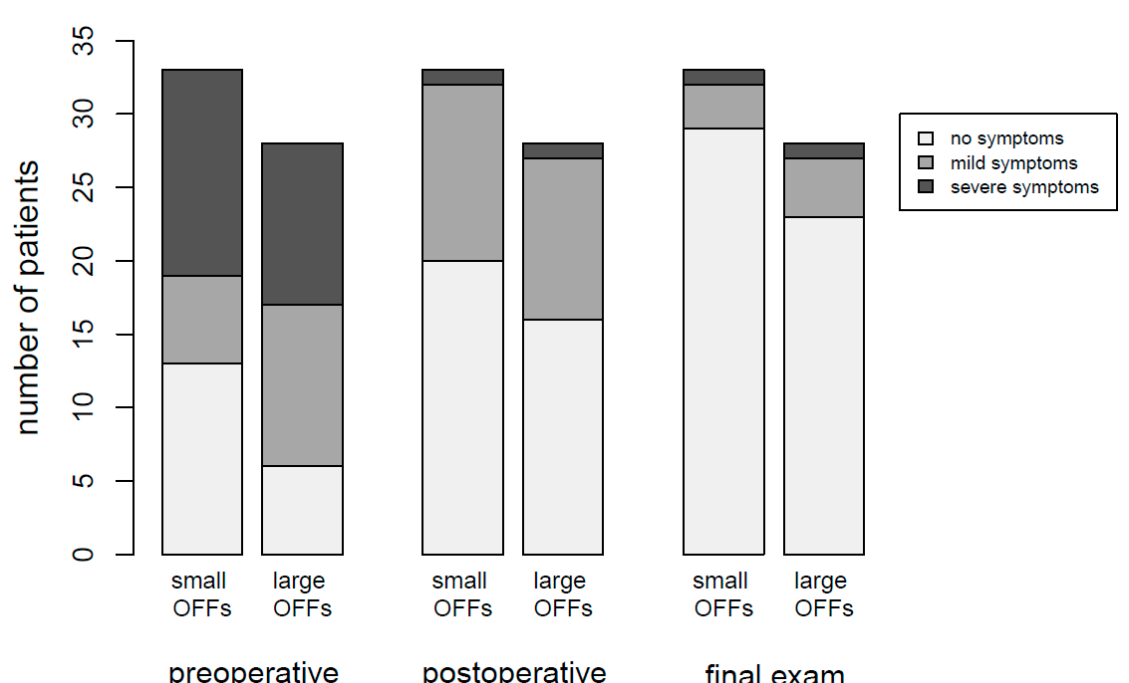
Stacked bar chart showing the results of orthoptic assessments at different timepoints.

The number of patients in category “severe impairment” decreased from 25 subjects (41.0%) in the preoperative evaluation to 2 patients (3.3%) in both the postoperative assessment as well as the last follow-up examination. The number of category “mild impairment” patients was 23 (37.7%) post surgery, and decreased to 7 subjects (11.5%) on final assessment. Details regarding both pre- and postoperative ophthalmologic and orthoptic assessments can be found in Table 1 and Table 2. In Table 3, results of long-term follow-up examinations up to one year post surgery are summarized. The results are illustrated in Figure 4.

The ophthalmologic findings were significantly different regarding preoperative, early postoperative and late postoperative examinations (χ^2^(2) = 55.552, *p* < 0.001). Post hoc analysis revealed statistically significant improvement of the ophthalmologic findings from pre- to early postoperative (*p* < 0.001), from early postoperative to late postoperative (*p* < 0.001) and from preoperative to late postoperative (*p* < 0.001) examinations.

In order to reveal potential differences between the clinical outcomes following surgery in small versus large OFFs, the number of patients in categories “no impairment”, “mild impairment”, and “severe impairment” were compared between both groups. A detailed breakdown of the respective numbers is provided in Table 4.

The Mann–Whitney U test did not show statistically significant differences between small and large OFFs, neither was such a difference demonstrated for the preoperative examinations (U = 423, *p* = 0.552), early postoperative examinations (U = 445.5, *p* = 0.788) or late postoperative examinations (U = 436, *p* = 0.549). For the post hoc power analysis using the Mann–Whitney U test for comparison of small versus large OFFs, a large effect size of Cohen’s d = 0.8 between the variables of interest was considered relevant. With the sample sizes of n = 33 and n = 28 for the two groups, the statistical power was calculated at 0.91 for discovering statistically significant differences at a significance level of α = 0.05. Consequently, the probability of a type II error was β = 0.09.

## 4. Discussion

Although undeniable progress in the management of orbital floor fractures has occurred over the past decades, there is still a lack of broad consensus as to which material(s) should be used to restore proper anatomy of the orbital floor in order to achieve best clinical results. This is also reflected by the still very high number of recent studies describing various approaches and materials to reconstruct the orbital floor, such as titanium meshes [27], partially absorbable meshes [28], bioresorbable implants [29,30], resorbable collagen membranes [31], polydioxanone foils [32], porous polyethylene sheets [33], bioactive glass S53P4 implants [34], auricular conchal grafts [35], rib bone grafts [36], or heterologous cortical bone [37].

With the emergence of new substances over the past decade, the management of patients under anticoagulants has become more challenging. Valuable information in this regard can be found in a recent review article [38]. Referring to our general protocol, we usually pause acetylsalicylic acid or clopidogrel 8 days preoperatively and recommence this medication 3 days postoperatively. DOACs (direct oral anticoagulants) are paused two days before surgery and are recommenced on the first postoperative day. In our patient collective, there were no subjects suffering from any congenital or acquired bleeding disorders. Neither intra- nor postoperatively were any bleeding complications noted. Following surgery, patients were advised to immediately report any symptoms of visual impairment which could potentially indicate intraorbital hemorrhage. Since the incidence of venous thromboembolic events in oral and maxillofacial surgery is very low ranging from 0.15% to 1.6% [39], we did not routinely administer thromboprophylaxis medication. There were no thromboembolic events pre-, intra- or postoperatively in any of our patients.

The ideal material to reconstruct or replace the bony floor of the orbit would have to be relatively thin (to avoid augmentation of the intraorbital volume), easy to crop and adjust to the specific size and form of the defect, rigid enough to support the whole of the orbital content, and highly biocompatible. Nonresorbable rigid materials, for example titanium meshes, bear the potential risk of resisting even strong forces in case of a second trauma which could eventually cause damage to the eyeball being prevented from displacing into the maxillary sinus as some sort of “self-protective” mechanism. This could be an argument against the use of rigid nonresorbable materials.

Polyglactin 910/polydioxanone (Ethisorb^®^) is a well-known material in neurosurgery, primarily used to repair dura mater defects. Apart from this main indication, it has also been used for OFF repair since many years, and this “off-label” use was reported in several studies [22,23,24,25]. In many aspects, Ethisorb^®^ represents a highly suitable material to restore proper anatomical outlines in the delicate area of the orbital floor. It is relatively thin, easy to crop, and highly biocompatible which is underlined by its widespread and well tolerated use in neurosurgery as mentioned above. Regarding rigidity, its properties certainly allow for restoration of small orbital defects. For larger defects, it could be argued that this relatively soft and bendable material would be too weak to provide sufficient support for the intraorbital content. It was therefore the main purpose of this study to compare the outcomes of both small and large OFFs reconstructed with the use of Ethisorb^®^.

To this avail, fractures classified as Jaquiéry I representing small OFFs (≤2 cm^2^) were compared to large OFFs including Jaquiéry categories II–IV. Statistical analysis of a total of 61 cases did not reveal any significant differences with regard to the final orthoptic assessment up to one year postoperatively. In this context, it could of course be argued that not detecting a significant difference does not necessarily mean absence of such a difference regarding the outcomes of both groups. In case the sample size is too small, an existing difference could not be revealed. Therefore, we undertook a power analysis using the aforementioned parameters.

Generally, a power level (1−β) of 0.8 is regarded as a statistically valid threshold in this context. Since our analysis revealed an even higher level of 0.91, we are confident that the number of patients included was sufficient to reveal a relevant difference between both groups, provided such a difference between small and large OFFs had been present.

Our analysis revealed a significant clinical improvement regarding both the pre- and early postoperative as well as the early postoperative and the late clinical examinations. In the light of these findings, long-term follow-up examinations appear to be sensible and useful if not mandatory.

In our department, great effort is made to reduce the bony fragments of the fractured orbital floor into the anatomically correct position prior to positioning the Ethisorb^®^ patch. The precise reduction of the bony fragments may play an important role for the functional outcome in these injuries. Following perfect reduction of the bony fragments, it is key to interlock the greater parts in order to achieve a stable result. In this case, the main function of the Ethisorb^®^ patch is not stabilization of the fracture but rather prevention of the soft tissue from getting caught in the fracture gap.

The advantages of Ethisorb^®^ are multifold: it is relatively cheap, it can be easily trimmed and placed straightforwardly once the fracture gap has been cleared from prolapsed soft tissue, it is thin and therefore does not lead to an increase in orbital volume, and it fully resorbs over time and does not leave a rigid foreign body behind which could potentially cause damage to the eyeball in case of a “second hit” injury.

Obviously, surgical repair using Ethisorb^®^ has its clear limitations, especially in large defects where the fracture fragments can not be reduced properly or where such a reduction is not stable. In our department, we always make a radiological assessment before surgery preselecting cases we believe could be treated with Ethisorb^®^. The final decision, however, is exclusively made intraoperatively. If the preconditions for surgical repair using Ethisorb^®^ are not met (i.e., no reduction of bony fragments possible, critical or no stability following fracture reduction, or large residual defect after bony reduction), a rigid material such as titanium is used.

Apart from these shortcomings of the Ethisorb^®^ patch itself, there are several limitations to this study which need to be discussed. First of all, this is a retrospective study and, thus, only routine documentation with its inherent limitations was available for analysis. Further, the total size of our sample was relatively small. One could be tempted to argue that the small sample size may be responsible for the fact that no difference in outcome between small and large defects was found. However, with regard to the power calculation as described above, the statistics should still be valuable. Further, the timing of the last follow-up examination may appear too early after surgery. In this context it is important to know that we do not usually invite patients for further follow-ups if no abnormalities are revealed at the second postoperative control visit. Besides, our hospital is situated in the middle of a large tourist area which means that many of our patients (especially tourists after skiing accidents) are followed up elsewhere when returning home. Finally, the statistical value of having only one class IV fracture to be included may seem doubtful. However, this single fracture was not analyzed separately but was included in the group “large OFF”.

The outcome of this study leads us to conclude that it is reasonable to advocate the use of Ethisorb^®^ for the repair of both small and large defects of the orbital floor. From our experience, this resorbable patch yields good clinical long-term results and should be considered a valuable treatment option for surgical repair of small and large fractures of the orbital floor.

## Figures and Tables

**Table 1:** Preoperative ophthalmologic and orthoptic assessment in 61 patients.

Jaquiéry Classification	Category I	Category II	Category III	Category IV	Total
orthoptically normal	15/33	3/20	3/7	0/1	21/61
double vision at >20°	14/33	14/20	4/7	1/1	33/61
permanent double vision	4/33	3/20	0/7	0/1	7/61
malposition of eyeball	6/33	4/20	1/7	1/1	12/61
impaired vision	1/33	1/20	0/7	0/1	2/61

**Table 2:** Postoperative ophthalmologic and orthoptic assessment in 61 patients.

Jaquiéry Classification	Category I	Category II	Category III	Category IV	Total
orthoptically normal	20/33	11/20	4/7	1/1	36/61
double vision at >20°	12/33	7/20	3/7	0/1	22/61
permanent double vision	1/33	2/20	0/7	0/1	3/61
malposition of eyeball	3/33	1/20	0/7	0/1	4/61
impaired vision	0/33	0/20	0/7	0/1	0/61

**Table 3:** Late ophthalmologic and orthoptic follow-up in 61 patients.

Jaquiéry Classification	Category I	Category II	Category III	Category IV	Total
orthoptically normal	29/33	16/20	6/7	1/1	52/61
double vision at >20°	3/33	3/20	1/7	0/1	7/61
Permanent double vision	1/33	1/20	0/7	0/1	2/61
malposition of eyeball	2/33	1/20	0/7	0/1	3/61
impaired vision	0/33	0/20	0/7	0/1	0/61

**Table 4:** Severity of pathological features in small and large OFFs pre- and postoperatively as well as on last follow-up examination.

	Small OFFs	Large OFFs	Total
**pre-OP**			
“no impairment”	13/33 (39.4%)	6/28 (21.4%)	19/61 (31.1%)
“mild impairment”	6/33 (18.2%)	11/28 (39.3%)	17/61 (27.9%)
“severe impairment”	14/33 (42.4%)	11/28 (39.3%)	25/61(41.0%)
**post-OP**			
“no impairment”	20/33 (60.6%)	16/28 (57.1%)	36/61 (59.0%)
“mild impairment”	12/33 (36.4%)	11/28 (39.3%)	23/61 (37.7%)
“severe impairment”	1/33 (3.0%)	1/28 (3.6%)	2/61 (3.3%)
**final exam**			
“no impairment”	29/33 (87.9%)	23/28 (82.1%)	52/61 (85.2%)
“mild impairment”	3/33 (9.1%)	4/28 (14.3%)	7/61 (11.5%)
“severe impairment”	1/33 (3.0%)	1/28 (3.6%)	2/61 (3.3%)
